# Role of nitric oxide in psychostimulant-induced neurotoxicity

**DOI:** 10.3934/Neuroscience.2019.3.191

**Published:** 2019-09-03

**Authors:** Valentina Bashkatova, Athineos Philippu

**Affiliations:** 1 Laboratory of physiology of reinforcement, P.K. Anokhin Institute of Normal Physiology, Moscow, Russia; 2 Department of Pharmacology and Toxicology, University of Innsbruck, Austria

**Keywords:** nitric oxide (NO), psychostimulant drugs, neurotoxicity, brain, striatum, electron paramagnetic resonance, amphetamine, Sydnocarb, NO synthase inhibitors, lipid peroxidation, antagonist NMDA glutamate receptor

## Abstract

In recent decades, consumption of psychostimulants has been significantly increased all over the world, while exact mechanisms of neurochemical effects of psychomotor stimulants remained unclear. It is assumed that the neuronal messenger nitric oxide (NO) may be involved in mechanisms of neurotoxicity evoked by psychomotor stimulants. However, possible participation of NO in various pathological states is supported mainly by indirect evidence because of its short half-life in tissues. Aim of this review is to describe the involvement of NO and the contribution of lipid peroxidation (LPO) and acetylcholine (ACH) release in neurotoxic effects of psychostimulant drugs. NO was directly determined in brain structures by electron paramagnetic resonance (EPR). Both NO generation and LPO products as well as release of ACH were increased in brain structures following four injections of amphetamine (AMPH). Pretreatment of rats with the non-selective inhibitor of NO-synthase (NOS) N-nitro-L-arginine or the neuronal NOS inhibitor 7-nitroindazole significantly reduced increase of NO generation as well as the rise of ACH release induced by AMPH. Both NOS inhibitors injected prior to AMPH had no effect on enhanced levels of LPO products. Administration of the noncompetitive NMDA receptor antagonist dizocilpine abolished increase of both NO content and concentration of LPO products induced by of the psychostimulant drug. Dizocilpine also eliminated the influence of AMPH on the ACH release. Moreover, the neurochemical and neurotoxic effects of the psychostimulant drug sydnocarb were compared with those of AMPH. Single injection of AMPH showed a more pronounced increase in NO and TBARS levels than after an equimolar concentration of sydnocarb. The findings demonstrate the crucial role of NO in the development of neurotoxicity elicited by psychostimulants and underline the key role of NOS in AMPH-induced neurotoxicity.

## 1. Introduction

Nitric oxide (NO) is a gaseous chemical messenger that participates in varied physiological functions [1],[2]. In line with the widespread expression of this pathway, NO participates in various brain functions. NO regulates the activity of neurotransmitter systems of the brain and the content of neuromediators in the extracellular space [3],[4]. The role of NO as a biological messenger is determined primarily by its physicochemical properties. It is a highly labile, short-living, reactive free radical [5],[6]. The conclusion that NO is a regulatory molecule possessing the properties of a biological messenger was a consequence of the development of numerous scientific fields, including the physiology and pharmacology of the cardiovascular system, toxicology, neurobiology, etc. In recent decades, the role of NO was shown in modeling of central nervous system diseases such as neurodegenerative disorders, stroke, epilepsy, neurotoxic damage [7]–[12].

The property of NO to cause a biological effect depends to a large extent on the small size of its molecule, its high reactivity, and its ability to diffuse in tissues, including the nervous system. This was the reason to call NO a retrograde messenger [13]. Recently, the importance of NO as a universal modulator in the brain has been postulated [2]. This compound is formed from L-arginine as a result of a two-step reaction of the enzymatic oxidation of its guanidine group to form an intermediate, NG-hydroxy-L-arginine. Several isoforms of NO-synthase (NOS) have been described: constitutive, permanently present in tissue and inducible. Thereby, activity of NOS is important for the manifestation of the physiological and neurochemical action of NO [14],[15].

Amphetamine-like psychostimulants, such as amphetamine (AMPH), 3,4-methylenedioxymethamphetamine (MDMA or Ecstasy), and methamphetamine (METH) are psychomotor stimulants that may cause addiction [16]. The mechanism of action of AMPH -like psychostimulants is associated with their ability to influence the monoaminergic systems of the brain. AMPH and its derivatives have a pronounced neurotoxic potential, which is manifested, in particular, in reducing the neuronal content of dopamine (DA), reducing the number of binding sites of the synaptic dopamine transporter [17], degeneration of dopaminergic terminals of the nigrostriatal system [18]–[21]. Recent reports claim that other brain neurotransmitters, such as glutamate and acetylcholine (ACH) are also involved in the mechanism of neurotoxicity evoked by psychostimulant [2],[22]–[24].

The precise mechanisms of AMPH-induced neurotoxicity remain unclear. Characteristic manifestations of the neurotoxic effect of AMPH and its derivatives, along with the depletion of intracellular DA and degeneration of neurons, are considered to be due to an generation of reactive oxygen species (ROS) and reactive nitrogen species (RNS [25]–[28]. Significant intensification of lipid peroxidation (LPO) processes in the brain was detected after a single injection of METH [29]. Increased generation of hydroxyl radicals as well as elevation of LPO products in rat striatum after AMPH administration has been observed [30]. Similar changes in hydroxyl radicals and LPO have been previously described during convulsions of various genesis as well as during global ischemia in rats [31]–[33]. It has been suggested that NOS inhibitor methyl N-nitro-L-arginine ester (L-NAME) [34], or neuronal NOS inhibitor (nNOS) 7-nitroindazole (7-NI) [35] prevent neurotoxic effects of AMPH-like psychostimulants in rats. L-NAME abolishes sensitization to AMPH after short-term and long-term withdrawal [36]. It has also been shown that treatment of newborns with a non-specific NOS inhibitor causes a long-term alternation in the content of NO in the brain with possible consequences for the transmission of DA [37]. Using immunocytochemistry and double in situ hybridization it has been demonstrated that serotonin neurones, which express NOS, are most vulnerable to toxicity induced by substitutes of AMPH such as 3,4-methylenedioxymethamphetamine and p-chloroamphetamine [38]. Furthermore, it has been shown that effects of AMPH on basal nNOS mRNA expression in neurons containing nNOS in the striatum depends on dose of the drug [39]. Similarly, the effect of AMPH on the increase in inducible NOS mRNA (iNOS) in highly aggressive proliferating immortalized microglia cells is also concenraion-dependent. [40]. Furthermore, it has been shown that repeated administration of MDMA elevates nNOS in the nigrostriatal system.

Aim of this review is to describe the involvement of NO and the contribution of LPO and ACH release in neurotoxic effects elicited by psychostimulant drugs. Furthermore, the effects of equimolar concentrations AMPH and the psychostimulant sydnocrab in on the levels of NO and LPO products in rat brain structures will be delineated.

## 2. Involvement of NO in neurotoxicity induced by amphetamine

NO levels were determined by electron paramagnetic resonance (EPR). This method allows to determine NO in vivo as a paramagnetic complex in organs and tissues, e.g. liver, heart, tumor etc. [41]–[43]. The method was slightly modified for detection of NO in brain structures of rats [44]. For our purposes, selective scavenger of NO diethyldithiocarbamate (Sigma, 500 mg/kg, i.p.) and a mixture of FeSO4 (37.5 mg/kg, s.c.) and sodium citrate (165 mg/kg, s.c.) were injected simultaneously and animals were decapitated after 30 minutes. The EPR spectra were recorded at 77 K using a Brucker ESR 300E spectrometer at a frequency of 9.33 kHz, hf-modulation frequency 0.5 mT, microwave power 20 mW and time constant 0.05 s. The concentration of trapped NO was calculated from the intensity of the third ultrafine splitting line of the resonance at g_⊥_ = 2.035.

We used the following two approaches to study the participation of NO in the development of neurotoxicity evoked by psychomotor stimulants.

### 2.1. Influence of NOS inhibitors and NMDA antagonist on NO and LPO in brain structures of rats treated with AMPH

**Figure 1.**
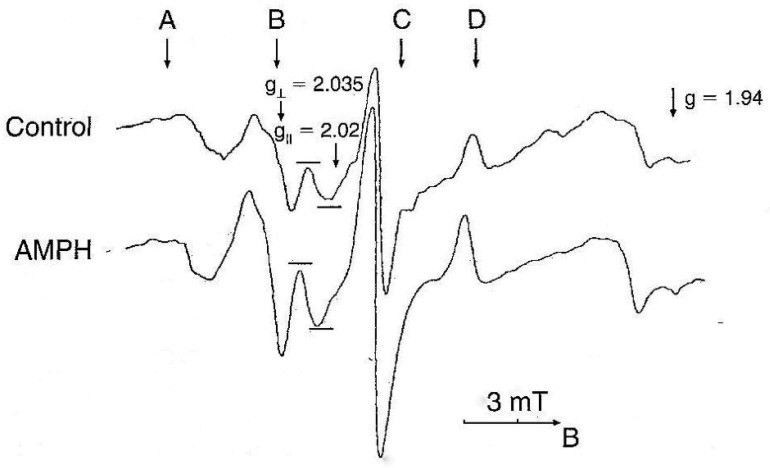
Typical EPR spectra of the cerebral cortex after administration of DETC and Fe citrate 30 min prior to decapitation. The signals at g_⊥_, g_||_ and g are due to NO-Fe-DETC and reduced iron-sulfur proteins in the mitochondrion respiratory chain. The arrows A, B, C and D indicate the position of components of ultrafine structure of EPR signals from Cu^2+^-DETC complexes at g_⊥_. Arrow direction of B extension of magnetic field [reproduced from Bashkatova et al., 1999].

Experiments were carried out on a male Sprague-Dawley rats (280–300 g) from the vivarium of University of Innsbruck. Protocols were approved by the Bundesministerium für Wissenschaft, Forschung und Kunst, Austria, Kommission für Tierversuchsangelegenheiten. Rats were injected with AMPH (Merck, Darmstadt, Germany) four times. The EPR signals of paramagnetic mononitrosyl MNIC–DETC complex registered in the brain cortex of rats are shown in Figure 1. The EPR signal, which represents NO, was enhanced following AMPH administration [45].

**Figure 2.**
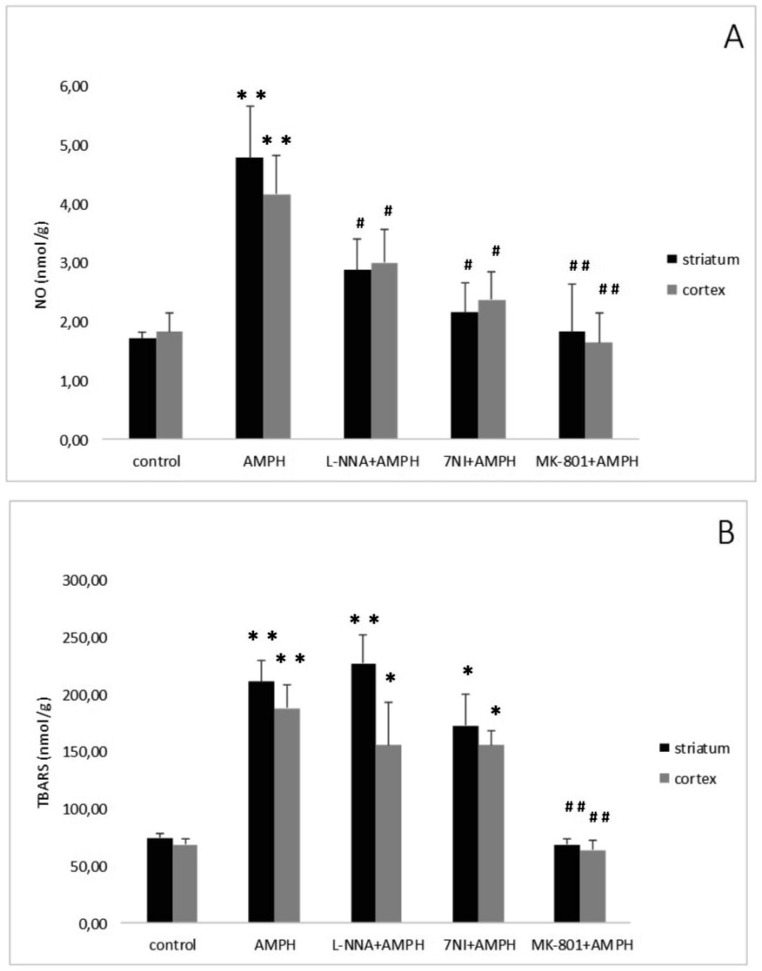
Effect of NOS inhibitors (L-NNA, 100 mg/kg, i.p., n = 7 and 7-NI, 50 mg/kg, i.p., n = 6) and NMDA antagonist dizocilpine (1 mg/kg, i.p., n = 6) injected 30 min prior the 1st AMPH injection (5 mg/kg, i.p., injected 4 times every 2 h, n = 6) on NO generation [A] and TBARS content [B] in striatum and cortex of rats. Data are the mean ± SEM. n = amount of rats/group. *P < 0.05, **P < 0.01 compared with the control (vehicle) group; ^#^P < 0.05, ^##^P < 0.01 compared with AMPH treated rats [to be published].

Repeated, systemic application of AMPH elevated striatal and cortical NO content (Figure 2A). Administration of the non-competitive NOS inhibitor N-nitro-L-arginine (L-NNA: Sigma, Deisenhofen, Germany) reduced but not abolished the elevation in NO levels evoked by AMPH. Similarly, the selective nNOS inhibitor 7-Nitroindazole (7-NI) (Sigma, Germany) significantly attenuated the AMPH induced NO generation [46]. The findings demonstrate that endogenous NO is implicated in neurotoxicity elicited by AMPH-like psychostimulants.

The interaction of NO with the neurotransmitter glutamate prompted us to study the possible role of this messenger in the pathophysiological mechanisms of AMPH-induced neurotoxicity. The inhibitor of NMDA glutamate receptors dizocilpine (MK-801, Research Biochemical International, Natick, MA, U.S.A.) was administered 30 min prior first AMPH injection. Dizocilpine abolished the rise of NO content induced by four injections of the psychostimulant (Figure 2A) [47]. This finding indicates that NMDA receptors mediate AMPH neurotoxicity and that, as already mentioned, NO is involved in this process.

NO might interact with other radicals, such as ROS. It is known that high doses of AMPH - like psychostimulants lead to an increase in the levels of hydroxyl radicals and LPO products in rat brain [48],[49]. Interaction of NO with ROS causes the generation of highly toxic products, in particular, peroxynitrite, which leads to damage and death of neurons [50]–[52]. However, the possible relationship of these processes is poorly understood. Intensity of LPO processes in brain areas was determined by measuring thiobarbituric acid reactive substances (TBARS). Briefly, tissue homogenate was mixed with sodium dodecyl sulfate, acetate buffer and thiobarbituric acid. After heating, the pigment was extracted with n-butanol-pyridine mixture and the absorbency was determined at 532 nm [53]. After the last injection of AMPH a more than two-fold increase in TBARS^−^level in the striatum and in the cortex was found [46] (Figure 2B). The NOS inhibitors (L-NNA or 7-NI) administered prior to AMPH failed the increase either striatal, or cortical content of TBARS (Figure 2B). Pretreatment with dizocilpine abolished AMPH-induced elevation of LPO levels in both brain areas (Figure 2B).

These findings are in accordance with our results carried out on the seizure models of rats [54],[55] and indicate that both the NO and NO-independent LPO are involved in the neurotoxicity caused by AMPH.

### 2.2. Influence of treatment with NOS inhibitors and NMDA antagonist on the ACH release in the nucleus accumbens of rats treated with AMPH

In our experiments the push-pull superfusion technique was used that makes it possible to determine quantitatively ACH released from their neurons in the synaptic cleft in distinct brain areas [47],[56]. For the determination of ACH release in the Nac the animals were anaesthetized with urethane, the head was fixed in a stereotaxic frame, and a push-pull cannula (outer tubing: outer diameter 0.83 mm, inner diameter 0.51 mm; inner tubing: outer diameter 0.31 mm, inner diameter 0.16 mm) was stereotaxically inserted through a hole in the skull into the Nac. The Nac was superfused with artificial cerebrospinal fluid which additionally contained neostigmine. The superfusate was continuously collected in time periods of 10 min. The superfusion rate was 20 µl/min. At the end of the experiment the rat was killed with an overdose of sodium phenobarbital and the brain was removed and immersed in formaldehyde solution. ACH was determined in the superfusate by high pressure liquid chromatography (HPLC) with electrochemical detection [56].

**Figure 3.**
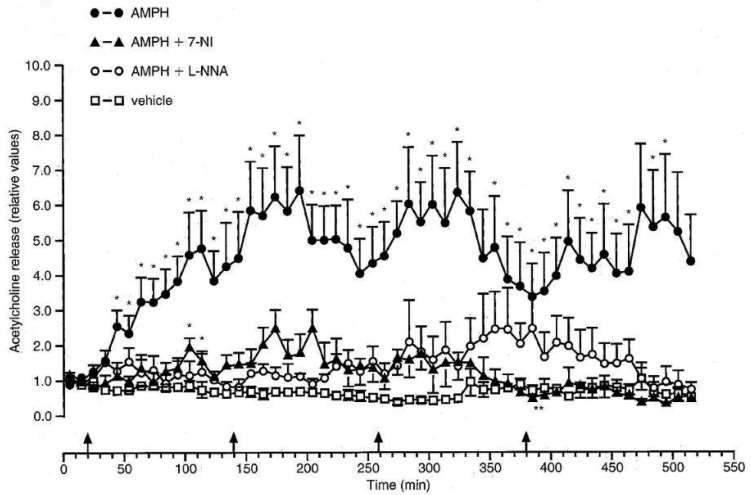
Effects of AMPH, of L-NNA and 7-NI on the release of ACH in the Nac. Arrows indicate injections of AMPH (5 mg/kg, i.p.), L-NNA (100 mg/kg, i.p.) and 7-NI (50 mg/kg, i.p.) were administered 30 min prior to the first injection of AMPH. The basal release rate in two samples preceding the first injection of AMPH was taken as 1. *P < 0.05 versus controls. Mean values ± s.e.m., n = 4–6 rats/group. Compounds were administered i.p. so as to make possible comparisons with other findings and to avoid interferences with other factors such as differing absortion rates [reproduced from Bashkatova et al., 1999].

**Figure 4.**
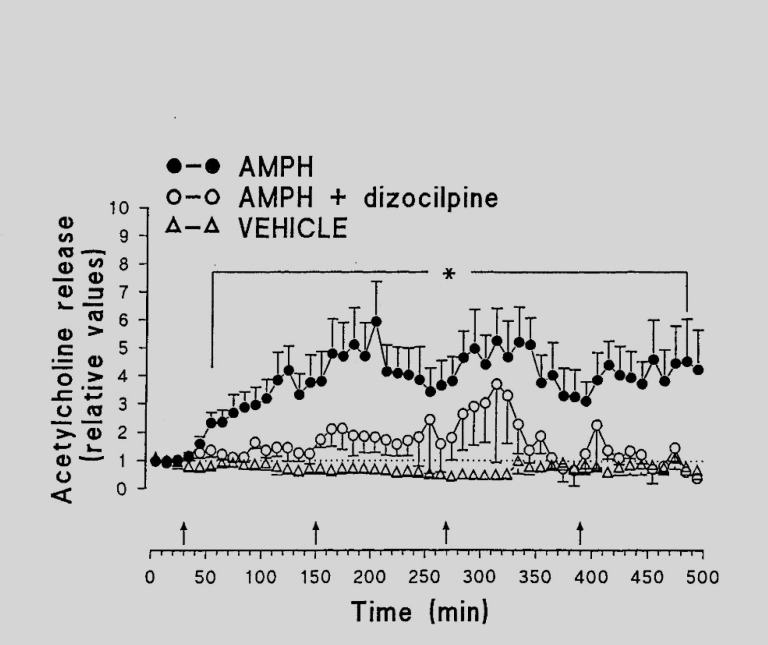
Effects of AMPH and dizocilpine (MK-801) on the release of ACH in the Nac. Arrows indicate injections of AMPH (5 mg/kg, i.p.). Dizocilpine (1 mg/kg, i.p.) was administered 30 min prior to the first injection of AMPH. The basal release rate in two samples preceding the first injection of AMPH was taken as 1. *P < 0.05 versus controls. Mean values ± s.e.m., n = 4–6 rats/group (reproduced from Kraus et al., 2002).

The mean basal output of ACH in the nucleus accumbens (NAc) was found to be 25.1 ± 9.1 fmol min^−1^. Four injections of the vehicle did not influence the release of ACH (Figure 3), while four repeated injections of AMPH led to a dramatic increase in the ACH release rates. The enhanced ACH release reached its maximum 40–120 min after administration of AMPH and persisted to the end of the experiment. NOS inhibitors (Figure 3) as well as the NMDA antagonist (Figure 4) almost completely prevented the increase of ACH release evoked by AMPH.

Very probably, the increase in ACH release following AMPH administration is due to the activation of nNOS. Moreover, the findings point to the crucial role of nNOS in the neurotoxic effects of AMPH. It is still controversial whether NO is functioning as protective agent against neurotoxic effects of AMPH [3],[57]. Our results underpin the idea that NO formation prevents neurotoxicity elucidated be AMPH [58].

It has been suggested that glutamatergic neurotransmission is involved in neurotoxicity elicited by AMPH [59]–[61]. As already mentioned (see 2.1.), the antagonist of NMDA receptor dizocilpine was very effective in reducing the ACH release caused by AMPH. Furthermore, dizocilpine prevented the increase of NO and LPO levels evoked by AMPH. Our results are in accordance with the observation that both NO and NMDA glutamate receptors are implicated in depressive conditions after amphetamine withdrawal [62]. Taken together, the data suggest that activation of NMDA receptors is necessary to induce AMPH neurotoxicity and to modify processes of neurotransmission within NAc.

## 3. Effects of sydnocarb on NO generation and LPO formation in brain areas of rats: comparison with amphetamine

In clinical practice, the administration of psychostimulants is limited due to a number of side effects. including their neurotoxic action. However, administration of psychostimulants is necessary for the treatment of a number of diseases of the central nervous system, especially in the case of attention deficit hyperactivity syndrome [63]–[65]. In this regard, the search for new drugs with psychostimulating action, but with less neurotoxicity than amphetamine, is one of the actual problems of modern pharmacology. Sydnocarb (*-phenylisopropyl) -N-phenylcarbamoyl sidnonimine), like other indirect dopaminomimetics, has a wide range of psychoactive properties [66]–[68]. Comparison of sydnocarb with METH has shown that the dysfunction of dopaminergic neurotransmission elicited by sydnocarb occurred more slowly and gradually than that of METH [69].

EPR technique was used to compare the effects of two psychostimulant drugs, AMPH and sydnocarb, at the equimolar doses (5 and 23.8 mg/kg, respectively) on the NO level in striatum and cortex of male Sprague Dawley rats (180–210 g) [70]. All experiments were performed in accordance with the French decree No. 87848/19 October 1987 and associated guidelines and the European Community Council directive 86/609/EEC/November 1986 (that corresponds to the recent Directive 2010/63/EU). AMPH greatly increased NO levels in the striatum and in the cortex of rats two hours after injection. Sydnocarb also increased NO content, however to a lesser extent than AMPH. Moreover, AMPH evoked more pronounced elevation of LPO products than sydnocarb in both brain areas. Hence, sydnocarb seems to be less neurotoxic than AMPH [70].

These findings are consistent with recent studies that AMPH has a significantly higher impact on the parameters of the stereotypical behavior of rats [71]. The maximum level of stereotypy (6 scores) was achieved within 2 hours after the first injection of the drug. Sydnocarb also caused motor stereotypy, but its intensity was significantly lower than that of AMPH (4 scores). Moreover, sydnocarb led to a slow and gradual increase of the parameters of dopaminergic dysfunction in comparison with AMPH [72]. Moreover, treatment with sydnocarb was accompanied by a less pronounced increase in formation of OH in comparison with that after AMPH administration [73]. It has been established that increased formation of free radicals induces LPO, which is considered to be one of indexes of neurotoxicity [74],[75]. Our findings demonstrate that AMPH as well as sydnocarb enhance NO generation and TBARS formation in rat brain. Finally, our results suggest that in striatum and cerebral cortex, AMPH, and to a lesser degree sydnocarb, may elicit neurotoxicity.

Taken together, these findings confirm that NO and ROS play important role in processes of neurotoxicity evoked by AMPH and sydnocarb. Furthermore, they point to the key role of neuronal NOS in AMPH- induced neurotoxicity and demonstrate the crucial role of NO in neurotoxicity induced by psychostimulant drugs.
